# Supplementary material for: Degrees of uncertainty: conformal deep learning for non-invasive core body temperature prediction in extreme environments
Source: Commun Eng. 2025 Nov 20;4:219. doi: 10.1038/s44172-025-00548-6 (PMC12727793; doi:10.1038/s44172-025-00548-6)
Supplement: Supplementary file 2 — Supplementary Information [file 44172_2025_548_MOESM2_ESM.pdf]

Supplementary Information

Degrees of Uncertainty: Conformal Deep Learning for Non-Invasive Core Body Temperature Prediction in Extreme Environments

Authors: Joel Strickland, Marco Ghisoni, Hannah Marshall, Thomas Whitehead, Bogdan Nenchev, Ben Pellegrini, Charles Phillips, Karl Tassenberg, Sarah Davey, Sandra Dorman, Joseph Sol, David Ferguson, Gareth Conduit

This file contains Supplementary Figures 1–7, Supplementary Table 1, and Supplementary References supporting the analyses and results described in the main manuscript.

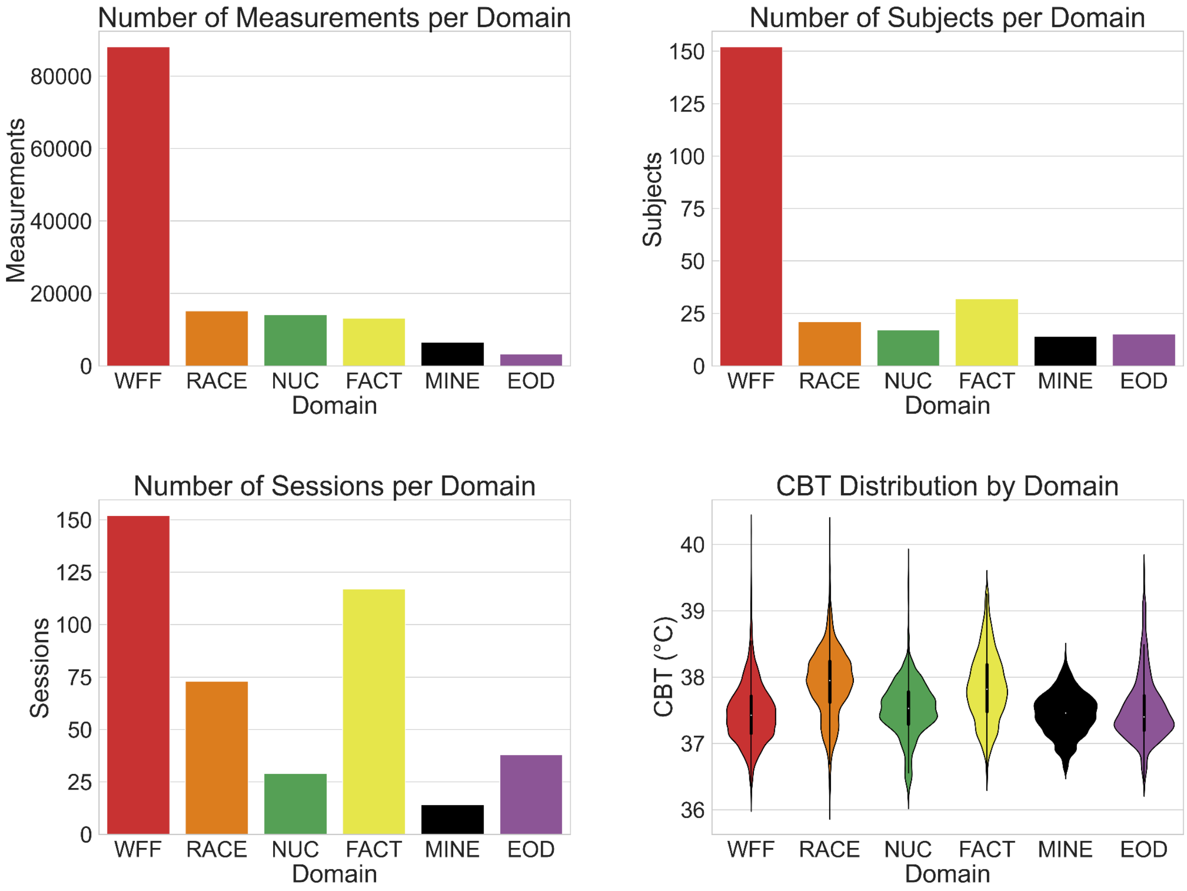

Supplementary Figure 1: Overview of dataset distribution across domains. Bar plots show the number of measurements, unique subjects, and sessions for each domain: wildland firefighters (WFF, red), race-car drivers (RACE, orange), nuclear power plant workers (NUC, green), factory workers (FACT, yellow), mine workers (MINE, black), and explosive ordnance disposal personnel (EOD, purple). Violin plots depict core body temperature (CBT) distributions for each domain, highlighting elevated CBT in WFF, RACE, and FACT domains due to task and environmental demands.

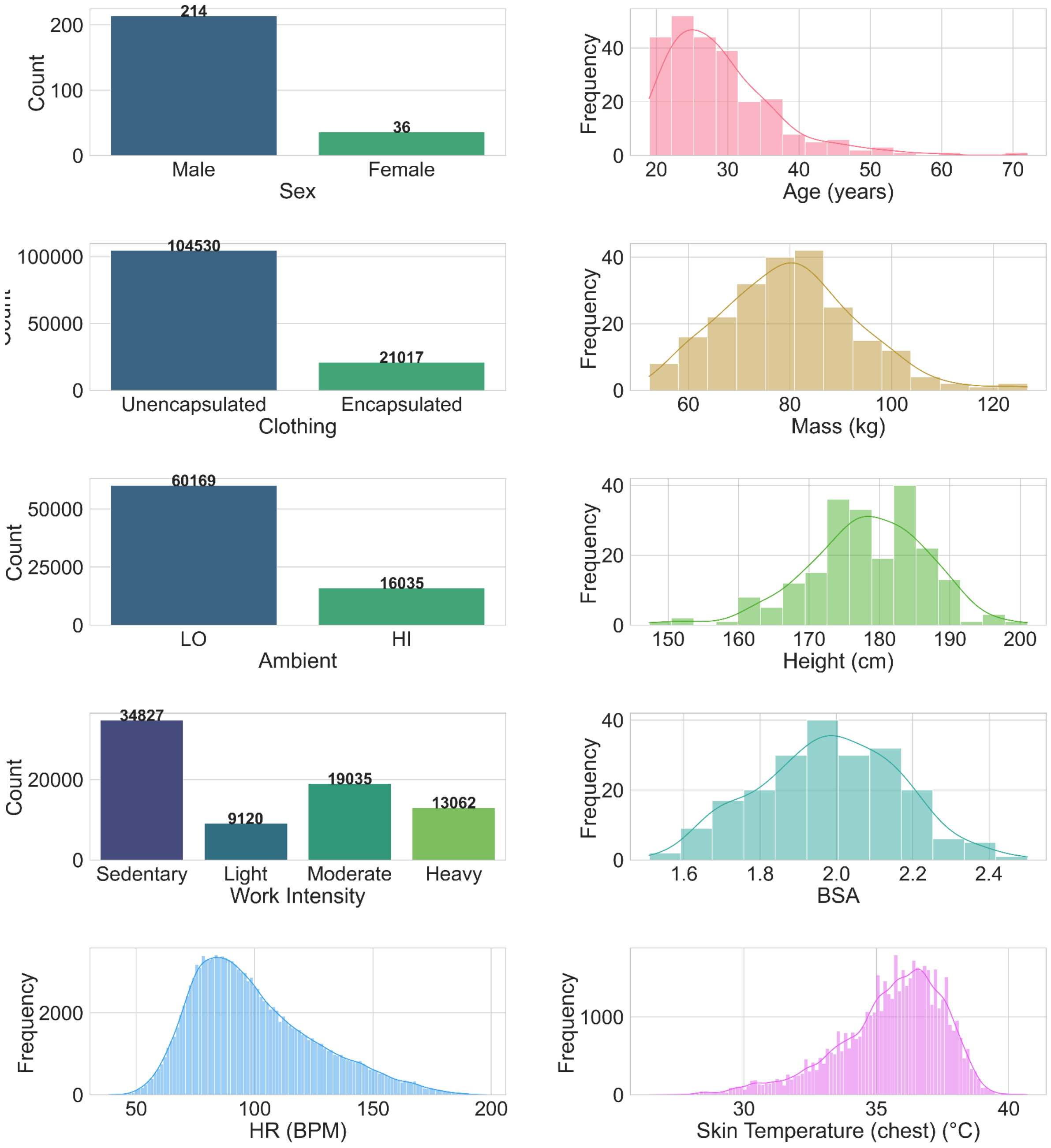

Supplementary Figure 2: **Distributions of variables within the dataset.** Histograms show distributions for physiological variables, including heart rate (with a peak at resting values and a long tail for intense activity), skin temperature (clustering between 32°C and 36°C, typical of hot compensable conditions[1]), body mass (70–90 kg), height (170–190 cm), body surface area (BSA; 1.8–2.2 m^2), and age (skewed toward younger individuals, 20–40 years). Bar plots summarize the proportion of encapsulated clothing conditions (21,017) versus unencapsulated (104,530) and ambient temperatures classified as low (<35°C; 60,169) or high (≥35°C; 16,035). These distributions highlight the diversity of operational scenarios and physiological responses in the dataset.

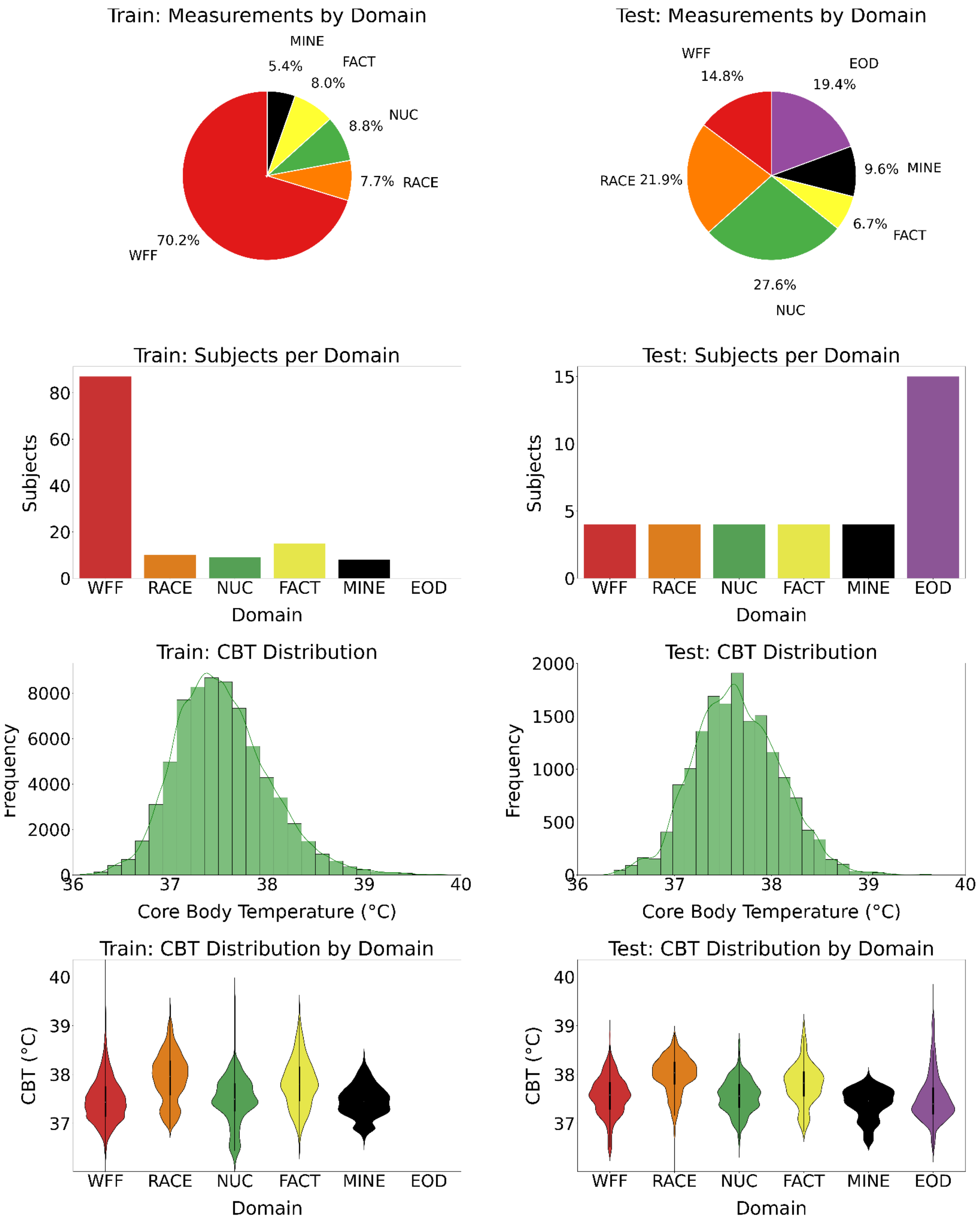

Supplementary Figure 3: Summary statistics for the training and test datasets, showing the distribution of domains, subjects, and core body temperature (CBT). This figure provides a comprehensive overview of the summary statistics across six domains in the training and test sets: wildland firefighters (WFF, red), race-car drivers (RACE, orange), nuclear plant workers (NUC, green), factory workers (FACT, yellow), mine workers (MINE, black), and explosive ordnance disposal technicians (EOD, purple). The first row features pie charts illustrating the proportional distribution of measurements across domains for the training and test datasets. In the second row, bar plots depict the number of unique subjects within

each domain, with WFF contributing the largest share in the training dataset. The third row presents histograms showing the overall distribution of core body temperature (CBT) values across the two datasets. The fourth row uses violin plots to offer a detailed view of CBT distributions stratified by domain, highlighting variations in temperature patterns unique to each domain. Together, these visualizations capture the dataset's composition, emphasizing domain representation, subject distribution, and CBT variability.

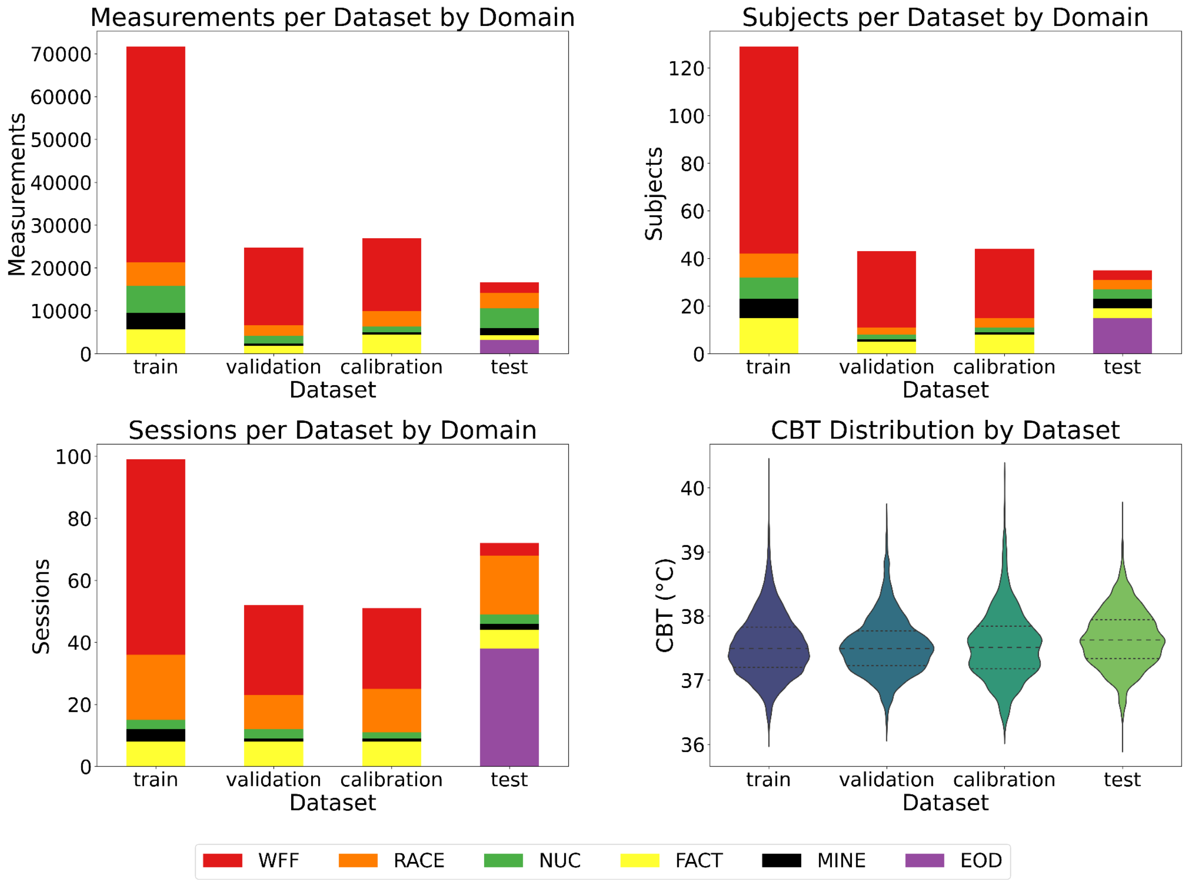

Supplementary Figure 4: Summary Statistics for Training, Validation, Calibration, and Test Datasets. This figure summarizes the distribution of measurements, subjects, sessions, and core body temperature (CBT) across the training, validation, calibration, and test datasets, categorized by domain. Domains are as follows: wildland firefighters (WFF, red), race-car drivers (RACE, orange), nuclear plant workers (NUC, green), factory workers (FACT, yellow), mine workers (MINE, black), and explosive ordnance disposal technicians (EOD, purple). While the WFF domain dominates the training, validation, and calibration datasets, the test set is more balanced. Bar plots show measurements, unique subjects, and sessions per dataset, while the violin plot illustrates CBT distributions, highlighting slight variations across datasets.

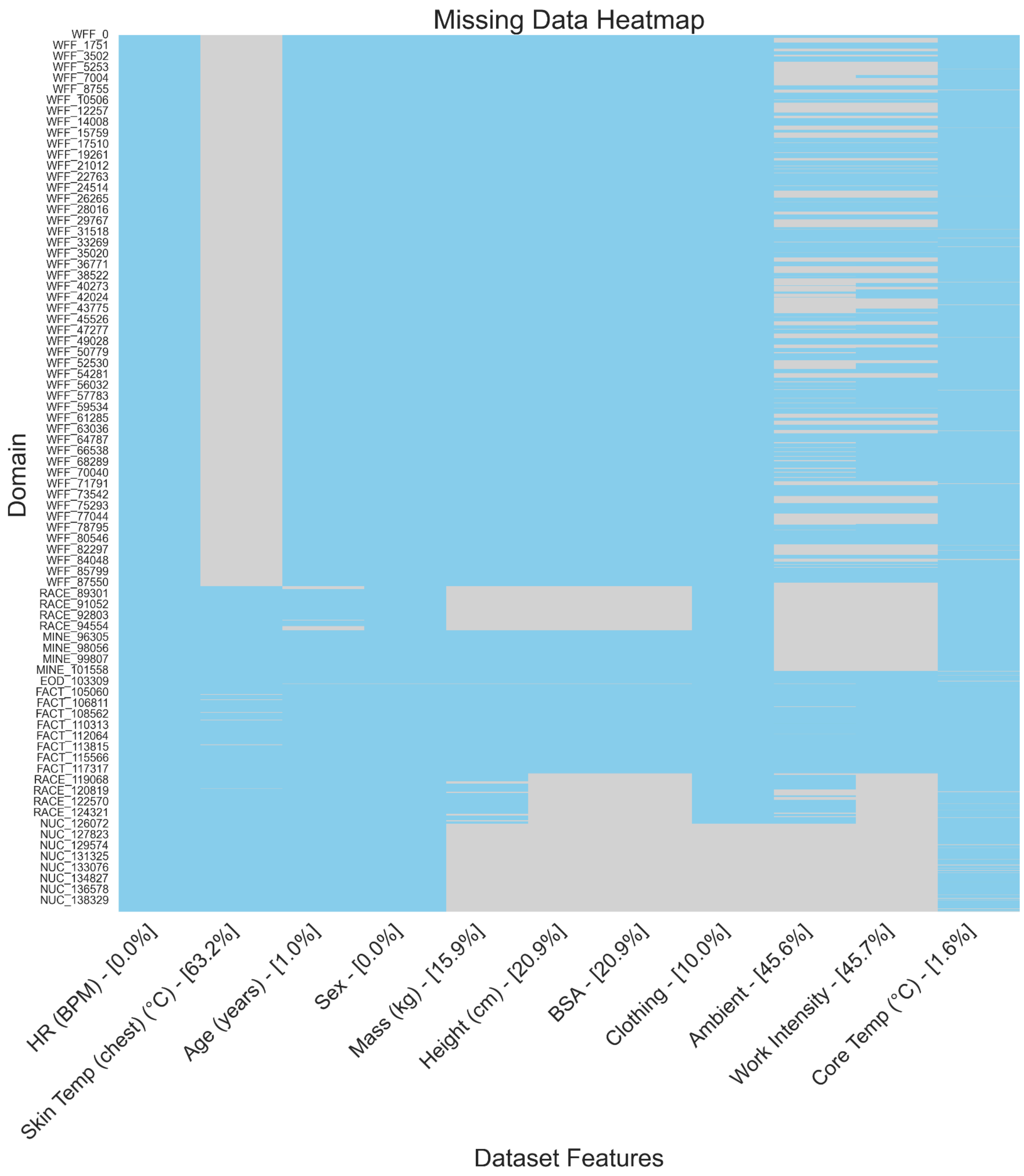

Supplementary Figure 5: Missing data heatmap for the variables within the dataset. The heatmap provides an overview of missing data across all variables. Light gray indicates missing data, while sky blue represents complete data. Rows correspond to specific domains—wildland firefighters (WFF), race-car drivers (RACE), nuclear power plant workers (NUC), factory workers (FACT), mine workers (MINE), and explosive ordnance disposal personnel (EOD)—and columns represent features in the dataset. Variables like heart rate (HR) are complete (0% missing), while others, such as skin temperature, exhibit substantial gaps (63%).

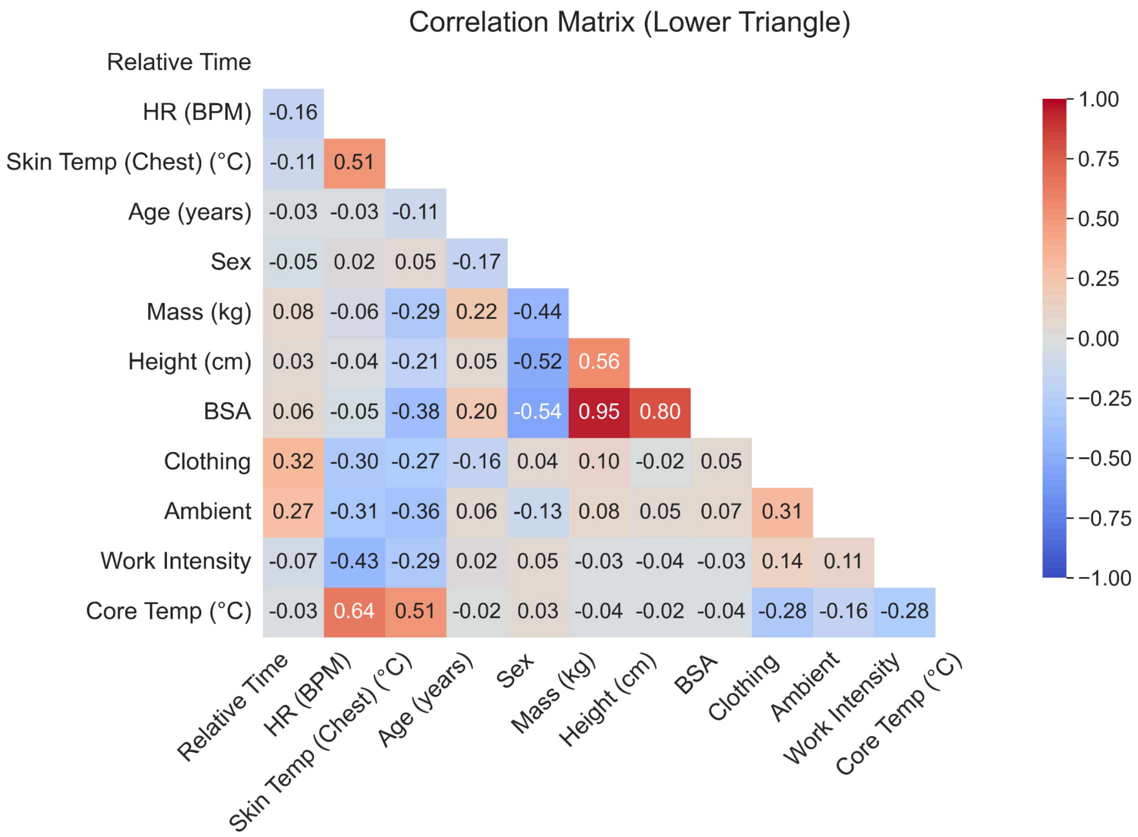

Supplementary Figure 6: Correlation matrix between predictive variables for core body temperature. The matrix illustrates the relationships among key physiological, demographic, and environmental features, with a color scale indicating correlation strength from -1 to 1. Strong positive correlations include body surface area (BSA) with height (0.95) and mass (0.80), as expected from their derived relationships. Heart rate is positively correlated with core body temperature (CBT; 0.64) and skin temperature (0.51), reflecting physiological responses to metabolic activity and thermoregulation during passive heating. Negative correlations include mass with age (-0.29) and height with age (-0.21), suggesting a younger, taller, and heavier population in the dataset. Work intensity is negatively correlated with heart rate (-0.43) and skin temperature (-0.29), indicating lower-intensity activities occur at lower heart rate and skin temperature values.

Supplementary Table 1: Model performance metrics (RMSE and R^2) for varying window lengths in the train, validation, calibration, and test sets. Root mean squared error (RMSE) and R^2 values are reported for train, validation, calibration, and test sets across temporal window lengths of 10, 20, 30, and 40 minutes. The 30-minute window consistently provided the best results, balancing model accuracy (lower RMSE) with stability (higher R2) across all datasets. This demonstrates the importance of selecting an optimal temporal resolution for robust model performance.

	**RMSE (°C)**				**R^2**			
**Window (mins)**	**Train**	**Validation**	**Calibration**	**Test**	**Train**	**Validation**	**Calibration**	**Test**
10	0.31	0.32	0.32	0.32	0.58	0.47	0.46	0.46
20	0.30	0.31	0.30	0.30	0.61	0.51	0.53	0.53
30	**0.30**	**0.31**	**0.29**	**0.29**	**0.62**	**0.52**	**0.57**	**0.57**
40	0.31	0.33	0.31	0.31	0.60	0.46	0.50	0.50

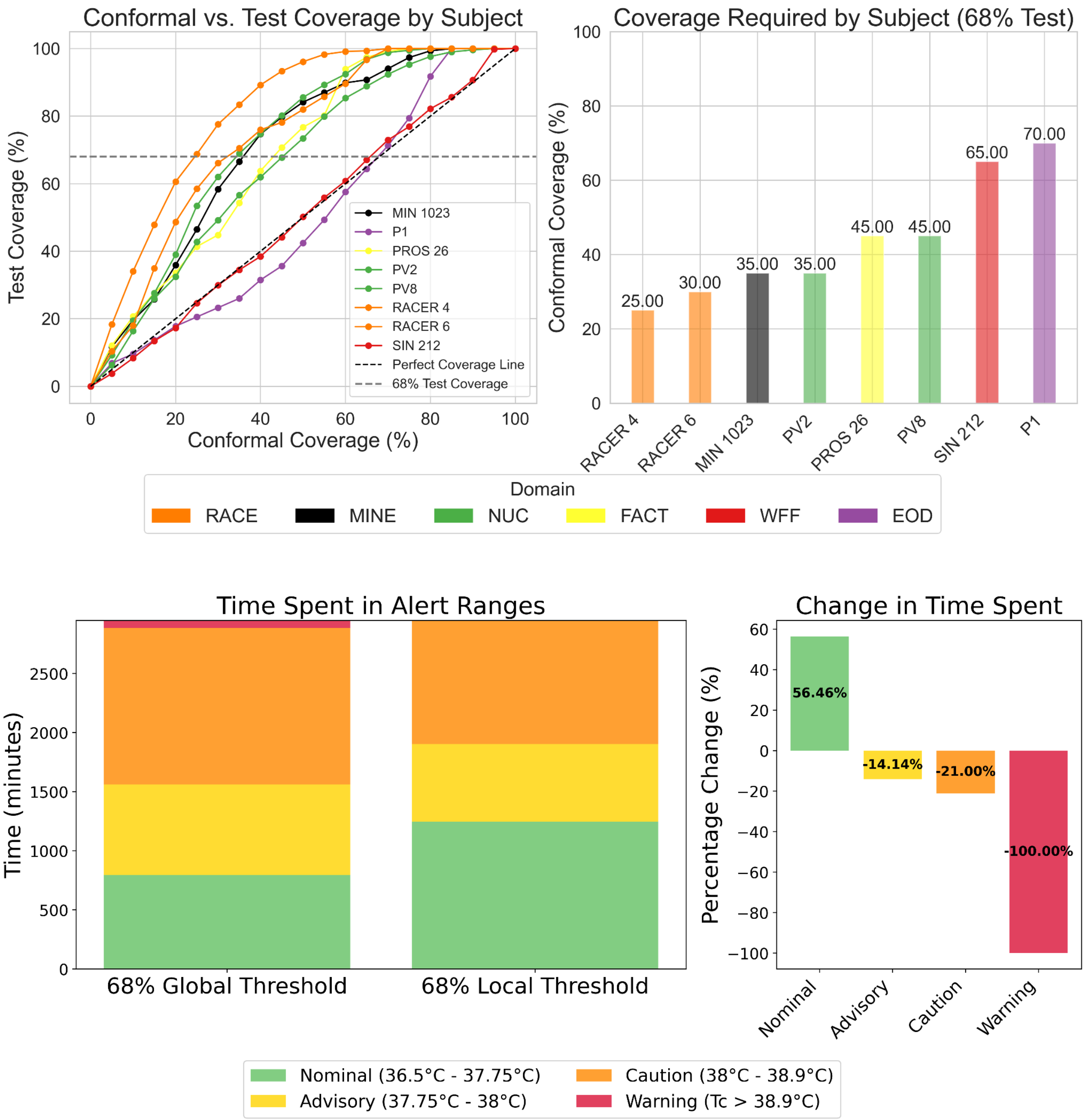

Supplementary Figure 7: Fine-tuning the SCOPE alert system for improved performance. *(Top panel)*: Conformal coverage percentages required to achieve 68% test coverage across eight different subjects from the test dataset, grouped by their respective domains: Wildland firefighters (WFF, red), race-car drivers (RACE, orange), nuclear plant workers (NUC, green), factory workers (FACT, yellow), mine workers (MINE, black) and explosive ordnance disposal technicians (EOD, purple). This highlights variability in predictive uncertainty across different environmental conditions. *(Bottom panel)*: Comparison of time spent in core body temperature ranges (nominal, advisory, caution, warning) for the same model with two different coverage adjustments: the standard 68% global coverage and a locally calibrated version that adjusts coverage based on observed real-world error. The stacked bar chart on the left shows the total time in each alert state, while the waterfall plot on the right highlights a 56.46% increase in Nominal time and a 100% reduction in Warning alerts, demonstrating the effectiveness of calibration in aligning alerts with actual CBT variation.

Supplementary References

1. Chou, T.H. and Coyle, E.F., 2023. Cardiovascular responses to hot skin at rest and during exercise. Temperature, 10(3), pp.326-357.
